# Flavonoids, Sterols and Lignans from *Cochlospermum vitifolium* and Their Relationship with Its Liver Activity

**DOI:** 10.3390/molecules23081952

**Published:** 2018-08-05

**Authors:** A. Berenice Aguilar-Guadarrama, María Yolanda Rios

**Affiliations:** Centro de Investigaciones Químicas, IICBA, Universidad Autónoma del Estado de Morelos, Avenida Universidad 1001, Col. Chamilpa, 62209 Cuernavaca, Morelos, Mexico; baguilar@uaem.mx

**Keywords:** *Cochlospermum vitifolium*, Cochlospermaceae, flavonoids, lignans, aromatic compounds, carotenoids, sterols, liver activity

## Abstract

The sterols β-sitostenone (**1**), stigmast-4,6,8(14),22-tetraen-3-one (**2**), β-sitosterol (**3**) and stigmasterol (**4**), the aromatic derivatives antiarol (**5**) and gentisic acid (**6**), the phenylpropanes coniferyl alcohol (**7**), epoxyconiferyl alcohol (**8**) and ferulic acid (**9**), the apocarotenoid vomifoliol (**10**), the flavonoids naringenin (**11**), 7,4′-dimethoxytaxifolin (7,4′-dimethoxydihydroquercetin, **12**), aromadendrin (**13**), kaempferol (**14**), taxifolin (dihydroquercetin, **15**), prunin (naringenin-7-*O*-β-d-glucoside, **16**), populnin (kaempferol-7-*O*-β-d-glucoside, **17**) and senecin (aromadendrin-7-*O*-β-d-glucoside, **18**) and the lignans kobusin (**19**) and pinoresinol (**20**), were isolated from the dried bark of *Cochlospermum vitifolium* Spreng (Cochlospermaceae), a Mexican medicinal plant used to treat jaundice, liver ailments and hepatitis C. Fourteen of these compounds were isolated for the first time from this plant and from the *Cochlospermum* genus. Compounds **3**–**4**, **6**–**7**, **9**–**11**, **13**–**17** and **20** have previously exhibited diverse beneficial liver activities. The presence of these compounds in *C. vitifolium* correlates with the use of this Mexican medicinal plant.

## 1. Introduction

The Cochlospermaceae family comprises seven genera: *Amoreuxia*, *Azeredia*, *Cochlospermum*, *Euryanthe*, *Lachnocistus*, *Maximilianea* and *Wittelsbachia*. In turn, the genus *Cochlospermum* (syn. *Maximilianea* and *Bixaceae*) is composed of 13 species of tropical trees ranging in height from 3 to 15 m, distributed in deciduous forests worldwide [1]. The bark and roots from the *Cochlospermum* species have been the most studied parts of these plants and only six of the 13 species of *Cochlospermum* have been chemically analyzed. Within *Cochlospermum gillivraei* the flavonoids apigenin, naringenin and afzelequin were found [2]; from *Cochlospermum gossypium* only carbohydrates were identified [3,4]; *Cochlospermum planchonii* biosynthesized the flavonoids miricetin, quercetin, aromadendrin and cianidin [5] and gallic acid, saponins, tannins, glycosides and carbohydrates [6]. From *Cochlospermum regium*, the gallic and ellagic acids, the flavonoid dihidrokaempferol-3-*O*-β-(6″-galloyl)-glucopyranoside and the lignans pinoresinol and excelsin have been isolated [7]. From *Cochlospermum tinctorium*, the triterpene arjunolic acid, along with tannins and carotenoids, β-bisabolene, 1-dodecanol, 1-hydroxy-3-octadecanone, 2-pentadecanone [8,9], alphitolic acid, cochloxantin and dihydrocochloxantin were identified [10]. Finally, the composition of the essential oils obtained from the leaves, root bark and root wood of *Cochlospermum vitifolium* has been established by GC/MS. The leaves’ essential oil consist of four major components: β-caryophyllene (46.5%), α-humulene (26.0%), β-pinene (10.6%) and α-pinene (4.8%), which, together, make up 87.9% of the total oil. The essential oil derived from the root’s bark is predominantly made up of β-bisabolene (29.3%), 1-hydroxy-3-hexadecanone (19.5%) and β-caryophyllene (8.2%), which corresponds to 57.0%. Furthermore, the root wood’s essential oil is composed of γ-muurolene (28.4%), 1-hydroxy-3-hexadecanone (16.2%), β-caryophyllene (11.6%), β-bisabolene (11.5%) and 2-dodecanone (6.3%), which represent 74.0% of the total essential oil [11]. The ethanol extracts from the root’s bark and wood are composed of gallic acid, the lignans excelsin and pinoresinol, the flavonoids naringenin and aromadendrin, and the sterols β-sitosterol, stigmasterol, 3-*O*-β-glycopyranosyl-β-sitosterol and 3-*O*-β-glycopyranosylstigmasterol while the root’s wood also contains 1-dodecanoyl-3,5-di(tetradecanoyl)benzene [11]. A second chemical analysis yielded the apocarotenoids cochloxanthin, dihydrocochloxanthin, vitixanthin and dihydrovitixanthin [12]. On the other hand, the plant’s flowers contain the flavonoids apigenin, naringenin and dihydroquercetin, and the carotenoids β-carotene, γ-carotene, lycopene, capsanthin, and zeaxanthin [13]. Finally, the stems contain naringenin and dihydroquercetin [14]. All of these studies indicate that the *Cochlospermum* species are characterized by the presence of sterols, flavonoids, carotenoids, apocarotenoids and lignans. 

*Cochlospermum vitifolium* (common name panicua, yellow rose or pongolote) is a medicinal tree which grows up to 5 m in height and is found from México to South America. It is characterized by its attractive yellow flowers and seed pods. This plant has been used in several countries due to its medicinal properties. In Cuba, for instance, a decoction of its leaves is used in the treatment of ulcers. In Costa Rica, the sap of the leaves is used to treat jaundice, and in Guatemala it is used due to its emmenagogue effects [15]. In some Mexican states, such as Morelos, Oaxaca, Puebla, and Veracruz, a decoction of its wood and leaves is drunk as an alternative treatment for liver and kidney ailments [16]. For example, in the state of Morelos, an infusion, prepared by boiling 10 g of its dried bark in 1 L of water, is used to treat hepatitis C, jaundice, liver diseases, diabetes, metabolic syndrome, and high blood pressure [17,18]. 

According to different studies, *Cochlospermum vitifolium*’s lethal dose 50 (LD_50_) in its lyophilized aqueous phase, obtained from the partitioned methanol extract, first with chloroform and then with ethyl acetate, was greater than 2000 mg/kg when administered intraperitoneally in mice [15]. The pharmacological analysis of the methanol extract from its dried bark demonstrated a decrease in noradrenaline induced vasoconstriction in rat aortic rings in a concentration and endothelium dependent manner (NO-cGMP system) [19]. The extract showed in vivo antihypertensive effects on spontaneously hypertensive rats [20] by inhibiting the [3H]-AT-II binding (angiotensin II AT1 receptor) by more than 50% [21]. In addition, hypoglycemic and antidiabetic effects were also seen in normoglycemic and STZ-nicotinamide-induced diabetic rats, both, in acute and subchronic models [22]. Additionally, the ethanol extract from the same part of the plant exhibited anti-inflammatory and immunomodulatory properties [23]. Finally, the dichloromethane extract was evaluated ex vivo using rat trachea rings to determine its relaxant activity against contractions induced by carbachol, showing a maximum effect at E_max_ = 106.58 ± 2.42% and an EC_50_ = 219.54 ± 7.61 μg/mL [24].

## 2. Results and Discussion

**Figure 1:**
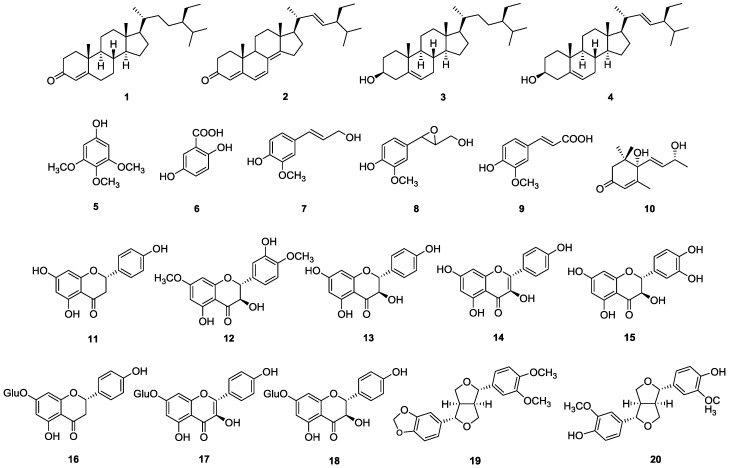
Chemical contents of the dried bark from *Cochlospermum vitifolium*.

During our ongoing phytochemical research from the dichloromethane extract of the dried bark of *Cochlospermum vitifolium*, the following metabolites were characterized: the sterols β-sitostenone (**1**), stigmast-4,6,8(14),22-tetraen-3-one (**2**), β-sitosterol (**3**) and stigmasterol (**4**), the aromatic derivatives antiarol (**5**), gentisic acid (**6**), coniferyl alcohol (**7**), epoxyconiferyl alcohol (**8**) and ferulic acid (**9**), the apocarotenoid vomifoliol (**10**), and the flavonoid naringenin (**11**). On the other hand, from the methanol extract two types of metabolites were identified: the flavonoids naringenin (**11**), 7,4′-dimethoxytaxifolin (7,4′-dimethoxydihydroquercetin, **12**), aromadendrin (**13**), kaempferol (**14**), taxifolin (dihydroquercetin, **15**), prunin (naringenin-7-*O*-β-d-glucoside, **16**), populnin (kaempferol-7-*O*-β-d-glucoside, **17**) and senecin (aromadendrin-7-*O*-β-d-glucoside, **18**); along with the lignans kobusin (**19**) and pinoresinol (**20**) (Figure 1). Vomifoliol (**10**) and naringenin (**11**) were the major constituents of the dichloromethane extract, while the flavonoids naringenin (**11**) and senecin (**18**) were isolated as the major components from the methanol extract. The structures for compounds **1**–**20** were established by the analysis of their ^1^H and ^13^C NMR (1D and 2D experiments) and MS spectra parameters and its comparison with those reported in the literature. The unequivocal ^1^H- and ^13^C- NMR assignments for compound **2** are reported here for the first time. 

Six of these 20 compounds (**3**, **4**, **11**, **13**, **15** and **20**) had been previously isolated from *Cochlospermum vitifolium*, however all other compounds were isolated here for the first time from this plant and from this genus. Compounds **1**–**20** belong to either the sterols; C_6_, C_6_–C_1_ and C_6_–C_3_ aromatic compounds; apocarotenoids; flavonoids and lignans groups, which are the most frequently identified compounds in this genus.

Previous studies have been conducted to demonstrate the antihypertensive [19,20,21,23], hypoglycemic and antidiabetic [22], immunomodulatory [23] and anti-inflammatory [24] effects of *Cochlospermum vitifolium* extracts. An exhaustive revision of the existing literature indicated that several of the compounds isolated in this research (Figure 1) can be associated to its popular use as different liver treatments. Due to the fact that this plant is broadly used as a treatment for jaundice, hepatitis C and other liver ailments in Mexican traditional medicine, its methanol extract was administrated at a dose of 100 mg/kg to bile duct-obstructed rats, to determine its hepatoprotective activity, showing a statistically significant decrease of serum glutamic-pyruvic transaminase (PGT, 45%) and alkaline phosphatase (APh, 15%) [19].

The importance of such activities lie in the fact that hepatic diseases (which comprise several conditions, such as: cirrhosis, hepatitis, alcoholic liver disease, non-alcoholic fatty liver disease, cholestatic and drug-induced liver diseases and liver cancer) are extremely high-priced in terms of human suffering, loss of productivity, and medical or hospital consultations. In fact, chronic liver diseases are the major cause of mortality worldwide. In 2013, 29 million people in Europe suffered from a chronic liver condition [25] and more than 30 million Americans had hepatic disease [26]. In China, liver diseases, viral hepatitis (predominantly hepatitis B virus), non-alcoholic fatty liver and alcoholic liver disease affect approximately 300 million people [27]. From a physiopathological perspective, most chronic liver diseases begin as an inflammatory process which evolves into focal fibrosis, and afterwards, to complete fibrosis of the gland (hepatic cirrhosis), which increases the risk of liver cancer. This leads to severe hepatic injury and ultimately to liver failure and other complications. 

Thirteen of the twenty compounds isolated from *Cochlospermum vitifolium* have previously exhibited in vitro and in vivo beneficial liver effects. β-Sitosterol (**3**) decreased hepatofibrosis [28], protecting against CCl_4_-induced hepatotoxicity [29] in animal models. Stigmasterol (**4**) induced apoptosis in hepatocarcimona (HepG2) cells being a potential antineoplastic therapeutic agent [30]. Gentisic acid (**6**) showed anti-inflammatory and antimutagenic properties, demonstrating protective effects against induced genotoxicity and hepatotoxicity [31]. On the other hand, coniferyl alcohol (**7**) had a moderated anti-hepatitis B virus (HBV) activity [32]. Ferulic acid (**9**) had an in vivo hepato-protective effect against the CCl_4_- and formaldehyde-induced hepatotoxicity [33] and also a capacity to inhibit the development of hepatic fibrosis by activation of Hepatic Stellate Cells (HSCs) in the presence of liver damage [34]. Vomifoliol (**10**) showed moderate activity against human hepatocarcinoma Hep3B cells [35]. Naringenin (**11**) had a potent lipid-lowering effect reducing the hepatic lipogenesis in rats and acting as an insulin sensitizer in vivo [36], thus, preventing rat liver damage caused by lead acetate, arsenic and high glucose. Furthermore, this same compound suppresses the metastatic potential of hepatocellular carcinoma [37]. Additionally, aromadendrin (**13**) possessed radical scavenging and activity against inflammatory, tumor and diabetic processes [38]. Kaempferol (**14**) had hepatoprotective effects in CCl_4_-, drug- and alcoholic-induced liver injury, constituting a promising therapeutic option for patients with atherosclerotic disease [39]. Taxifolin (**15**) had antioxidant and cytoprotective effects that prevent and help treat fulminant hepatitis and hepatitis caused CCl_4_ [40]. This natural flavonoid is licensed as Silymarin (Legalon^®^), a drug used for the treatment of toxic liver damage, chronic inflammatory liver disease and liver cirrhosis. Prunin (**16**) showed activity against the hepatitis B (HBV) virus with an IC_50_ of 41.59 μM [41]. Administered at a dose of 25 mg/kg, populnin (**17**) exhibited in vivo hepatoprotective effects against CCl_4_- and d-GalN-induced hepatotoxicity, preventing the development of hepatic lesions [42]. Finally, at 50 and 100 mg/kg, the lignane pinoresinol (**20**) showed hepatoprotective effects improving CCl_4_-induced liver injury [43]. All these liver beneficial effects from the compounds isolated from *Cochlospermum vitifolium* directly correlate with its traditional use in Mexican medicine.

## 3. Materials and Methods

### 3.1. General Procedures

Compounds **1**–**20** were purified by successive open column chromatography (CC) using silica gel (70–230 and 230–400 mesh, Sigma-Aldrich, Toluca, México). The isolation procedures and purity of compounds were monitored by thin layer chromatography (TLC) using precoated silica gel 60 F_254_ aluminium sheets, visualizing with UV-light and subsequently spraying the plates with (NH_4_)_4_Ce(SO_4_)_4_ in 2 N H_2_SO_4_ (Sigma-Aldrich, Toluca, México). All ^1^H-, ^13^C- and 2-D NMR experiments were performed in CDCl_3_ on a Varian Unity 400 spectrometer (Varian, Inc., Palo Alto, CA, USA) equipped with a 5 mm inverse detection pulse field gradient probe at 25 °C, at 400 MHz for ^1^H-NMR and 100 MHz for ^13^C-NMR. Chemical shifts were referenced to tetramethylsilane as an internal standard.

### 3.2. Plant Material

The wood of *Cochlospermum vitifolium* was collected from “Sierra de Huautla” (20°26′10″ N, 99°05′42″ W, 1915 m above sea level), Morelos, México, in October 2011, and identified by Dr. Rolando Ramírez Rodríguez, Centro de Investigación en Biodiversidad y Conservación-UAEM. A voucher specimen (number 14628) was deposited at HUMO Herbarium from the Universidad Autónoma del Estado de Morelos, México.

### 3.3. Extraction and Isolation

Throughout three months the wood of this plant was dried at room temperature. The dried and ground wood (1.65 kg) was extracted with CH_2_Cl_2_ and MeOH. These extracts were dried under a vacuum to render 11.9 g (0.72% yield) and 25.8 g (1.56% yield) of residue, respectively. 

Fractionation of the CH_2_Cl_2_ extract by open CC (silica gel, 70–230 mesh; 5 cm i.d. × 20 cm) was performed with a step gradient of *n*-hexane-acetone 100:0 to 0:100, collecting 330 fractions of 50 mL each. Based on TLC analysis, these fractions were pooled into nine groups, namely G-1 (fractions 1–70, *n*-hexane 100%), G-2 (fractions 71–76, 722 mg, *n*-hexane:acetone 95:5), G-3 (fractions 77–81, *n*-hexane:acetone 95:5), G-4 (fractions 82–89, 131 mg, *n*-hexane:acetone 95:5), G-5 (fractions 90–166, 780 mg, *n*-hexane:acetone 95:5), G-6 (fractions 167–188, 620 mg, *n*-hexane:acetone 9:1), G-7 (fractions 189–265, 639 mg, *n*-hexane:acetone 8:2), G-8 (fractions 266–324, 540 mg, *n*-hexane:acetone 1:1) and G-9 (fractions 325–335, acetone 100%). G-1 was made up of fatty acids, G-3 of triglycerides and G-9 of resins. The rest of the groups were subjected to column chromatography using silica gel 70–230 mesh. G-2 (2.0 cm i.d. × 30 cm, eluent *n*-hexane 100% to *n*-hexane:acetone 8:2) yielded 45 fractions of 40 mL. Fractions 23–33 (255 mg) were subjected to a second column chromatography (1.0 cm i.d. × 30 cm, eluent *n*-hexane:AcOEt 99:1) obtaining 91 fractions of 20 mL to yield β-sitosterone (**1**, 58 mg, 0.0035% with respects dry weigh of plant material). G-4 (1.5 cm i.d. × 15 cm, eluent *n*-hexane:AcOEt 99:1 to *n*-hexane:AcOEt 97:3) yielded 132 fractions of 50 mL which rendered stigmast-4,6,8(14),22-tetraen-3-one (**2**, 71 mg, 0.0043%), coniferyl alcohol (**7**, 21 mg, 0.0013%) and ferulic acid (**9**, 27 mg, 0.0016%). G-5 (2.0 cm i.d. × 30 cm, eluent *n*-hexane:AcOEt 97:3 to *n*-hexane:AcOEt 9:1) yielded 180 fractions of 50 mL resulting in the isolation of a 7:3 mixture of β-sitosterol (**3**) and stigmasterol (**4**, 280 mg, 0.0109%). G-6 (2.0 cm i.d. × 30 cm, eluent *n*-hexane:AcOEt 9:1 to *n*-hexane:AcOEt 7:3) yielded 77 fractions of 50 mL resulting in vomifoliol (**10**, 94 mg, 0.0056%). G-7 (2.0 cm i.d. × 30 cm, eluent *n*-hexane:AcOEt 9:1) yielded 50 fractions of 50 mL obtaining antiarol (**5**, 31 mg, 0.0018%) and gentisic acid (**6**, 29 mg, 0.0017%). Finally, G-8 (2.0 cm i.d. × 30 cm, *n*-hexane:acetone 8:2) yielded 187 fractions of 50 mL. Fractions 104–167 (280 mg) were subjected to a second column chromatography (1.0 cm i.d. × 30 cm, eluent *n*-hexane:AcOEt 6:4) resulting in 248 fractions of 20 mL to yield naringenin (**11**, 102 mg, 0.0061%) and epoxy-coniferyl alcohol (**8**, 36 mg, 0.0022%).

Fractionation of the MeOH extract by open CC (silica gel, 100–230 mesh; 5 cm i.d. × 30 cm) was performed with a step gradient of *n*-hexane-acetone 100:0 to 0:100, collecting 268 fractions of 50 mL each. These fractions were pooled into three groups: MG-1 (fractions 1–93, *n*-hexane to *n*-hexane:acetone 6:4), MG-2 (fractions 94–135, 2.2 g, *n*-hexane:acetone 1:1) and MG-3 (fractions 136–268, 3.29 g, *n*-hexane:acetone 1:1 to acetone). MG-1 was a complex mixture including compounds **1**, **3**, **4**, **11** and aromadendrin (**13**); MG-2 (3.0 cm *i.d.* × 30 cm, eluent *n*-hexane:AcOEt 85:15 to *n*-hexane:AcOEt 75:25) yielded 250 fractions of 50 mL which resulted in the purification of naringenin (**11**, 214 mg, 0.0129%); and MG-3 (4.0 cm *i.d.* × 30 cm, eluent *n*-hexane:AcOEt 7:3 to acetone) yielded 320 fractions of 50 mL. Fractions 33–51 (181 mg) were subjected to a second column chromatography (1.5 cm *i.d.* × 20 cm, eluent with *n*-hexane:AcOEt 7:3) collecting 56 fractions of 20 mL to yield naringenin (**11**, 41 mg, 0.0024%) and 7,4′-dimethoxy-taxifolin (**12**, 21 mg, 0.0013%). Fractions 77–83 (52 mg) were subjected to a preparative TLC (2 mm × 20 cm, eluent *n*-hexane:AcOEt 7:3 twice) to yield 30 mg (0.0018%) of a 6:4 mixture of **13** and kaempferol (**14**). Fractions 100–111 (49 mg) were subjected to a preparative TLC (2 mm × 20 cm, eluent *n*-hexane:AcOEt 7:3 twice) to yield kobusin (**19**, 19 mg, 0.0011%) and pinoresinol (**20**, 17 mg, 0.0010%). Fractions 145–182 (93 mg) were subjected to a preparative TLC (2 mm × 20 cm, eluent with CH_2_Cl_2_:MeOH 93:7) to yield taxifolin (**15**, 26 mg, 0.0016%). Finally, fractions 289–309 (691 mg) were subjected to a second column chromatography (2.0 cm *i.d.* × 30 cm, eluent with CH_2_Cl_2_:MeOH 9:1) collecting 140 fractions of 20 mL to yield prunin (**16**, 32 mg, 0.0019%), and 59 mg (0.0035%) of a 45:55 mixture of **16**, populnin (**17**), and senecin (**18**, 47 mg, 0.0028%). 

*Stigmast-4,6,8(14),22-tetraen-3-one* (**2**). ^1^H-NMR (CDCl_3_): δ 6.61 (1H, d, *J* = 7.2, H-7), 6.04 (1H, d, *J* = 7.2, H-6), 5.74 (1H, s, H-4), 5.27 (1H, dd, *J* = 15.2, 6.8, H-22), 5.20 (1H, dd, *J* = 15.2, 7.6, H-23), 2.49 (1H, m, H-2a), 2.57 (1H, m, H-2b), 2.51 (1H, m, H-15b), 2.47 (1H, m, H-15a), 2.16 (1H, m, H-20), 2.15 (1H, m, H-9), 2.10 (1H, m, H-12a), 2.03 (1H, m, H-1a), 2.54 (1H, m, H-1b), 1.88 (1H, m, H-24), 1.83 (1H, m, H-16b), 1.53 (1H, m, H-16a), 1.51 (1H, m, H-25), 1.32 (1H, m, H-12b), 1.30 (1H, m, H-17), 1.06 (3H, d, *J* = 6.4, H-21), 1.00 (3H, s, H-19), 0.96 (3H, s, H-18), 0.93 (3H, d, *J* = 6.8, H-26), 0.85 (3H, d, *J* = 6.8, H-27), 0.84 (3H, t, *J* = 6.8, H-29), 0.78 (2H, m, H-28). ^13^C-NMR (CDCl_3_): δ 199.76 (s, C-3), 164.67 (s, C-5), 156.35 (s, C-14), 135.25 (d, C-22), 134.28 (d, C-7), 132.78 (d, C-23), 124.71 (d, C-6), 124.66 (s, C-8), 123.23 (d, C-4), 55.95 (d, C-17), 44.57 (d, C-9), 44.24 (s, C-13), 43.13 (d, C-24), 39.55 (d, C-20), 37.02 (s, C-10), 35.84 (t, C-12), 34.37 (t, C-1, C-2), 33.34 d, C-25), 27.98 (t, C-16), 25.63 (t, C-15), 22.98 (t, C-11), 21.48 (q, C-21), 20.25 (q, C-27), 19.92 (q, C-18), 19.21 (q, C-28), 17.90 (q, C-26), 16.91 (q, C-19), 14.38 (q, C-29). 

## 4. Conclusions

*Cochlospermum vitifolium* biosynthesizes among other compounds the sterols **3** and **4**, the aromatic compounds **6**, **7** and **9**, the apocatrotenoid **10**, the flavonoids **11** and **13**–**17**, and the lignan **20**, which have demonstrated beneficial activity to alleviate different liver diseases. The presence of these compounds in the plant agrees with its traditional use in Mexican medicine and some are even included in commercial pharmaceutical formulations used in the treatment of hepatopathies. Their presence within *Cochlospermum vitifolium* extracts indicates that this plant could be an active hepatoprotective agent. However, the human consumption of this plant must be subjected to toxicity, pharmacodynamic and pharmacokinetic studies to determine how it can be a health contributor. 

The isolated metabolites in this study and the chemical composition previously reported for *Cochlospermum vitifolium* agree with the metabolic content within other *Cochlospermum* species. The flavonoids, sterols, carotenoids, apocarotenoids and lignans isolated here have chemotaxonomic significance within this genus. This is the first report of compounds **1**–**2**, **5**–**10**, **12**, **14**, **16**–**19** from *Cochlospermum vitifolium*.
